# The second cutaneous anthrax infection diagnosed by metagenomic next-generation sequencing: A case report

**DOI:** 10.1097/MD.0000000000036921

**Published:** 2024-01-19

**Authors:** Cuiying Zheng, Jiaqing Ye, Minghui Song, Yumei Guo, Weili Gao, Jiahao Hao, Zhongjun Feng, Lijie Zhang

**Affiliations:** a Department of Clinical Laboratory, The Third Hospital of Hebei Medical University, Hebei, China; b Hebei Key Laboratory of Intractable Pathogens, Shijiazhuang Center for Disease Control and Prevention, Hebei, China.

**Keywords:** anti-infection therapy, *Bacillus anthracis*, *Cutaneous anthrax*, mNGS

## Abstract

**Rationale:**

Anthrax is a severe zoonotic infectious disease caused by *Bacillus anthracis*. Most reported cases were traditionally diagnosed through culture and microscopy. We reported here the second case of cutaneous anthrax diagnosed by metagenomic next-generation sequencing (mNGS).

**Patient concerns:**

A 63-year-old man had a history of contact with an unwell sheep, developing local redness and swelling on wrist. The dorsal side of the left hand and forearm, with tension blisters on the back of the left.

**Diagnosis:**

*B anthracis* was detected from culturing and mNGS of tension blisters.

**Interventions:**

On the second day of admission, the patient was administered 3.2 million units of penicillin every 6 hours, and isolated and closely observed.

**Outcomes:**

The patient improves and is discharged.

**Lessons:**

Traditional bacterial cultures are time-consuming, while mNGS offers the advantage of accurate, quick, high-throughput, unbiased sequencing of all genetic material in a sample, which is a good technical tool for assisting in the diagnosis of rare pathogen infections.

## 1. Introduction

Anthrax occurs worldwide, however, due to widespread vaccination and extensive control measures in animals, animal and human anthrax has almost disappeared in developed countries. However, in developing countries, the disease is still prevalent in certain areas. The annual number of global cases is estimated to be between 20,000 and 100,000, predominantly in Africa, the Middle East, South America, Central Asia and Haiti.^[1]^ A total of 1244 human anthrax cases were reported in China from 2018 to 2021, mainly distributed in the west and northeast of China, such as Sichuan Province, Gansu Province, Qinghai Province, Inner Mongolia Autonomous Region, and Xinjiang Uygur Autonomous Region.^[2]^ *Bacillus anthracis* spores are highly resistant. Clinical manifestations range from mild to severe, with a 20% mortality rate if not treated early enough.^[3–5]^ But mortality rates for adequately treated anthrax range from <2% for cutaneous anthrax.^[6]^

A 63-year-old man was diagnosed with cutaneous anthrax following contact with an infected sheep, developing local redness and swelling on the dorsal side of the left hand and forearm, with tension blisters on the back of the left wrist. *B anthracis* was detected from culturing and metagenomic next-generation sequencing (mNGS) of tension blisters. After isolation and penicillin treatment, the patient was discharged with scabs. Most case reports in the literature primarily rely on culture and microscopy for diagnosis, with only one prior instance documented using mNGS for anthrax diagnosis.^[7]^ This study represents the second case in which mNGS was employed for anthrax diagnosis, identified through a search on the NCBI database. To raise awareness, we report the first case of cutaneous anthrax treated in our hospital.

## 2. Case reports

**Figure 1.**
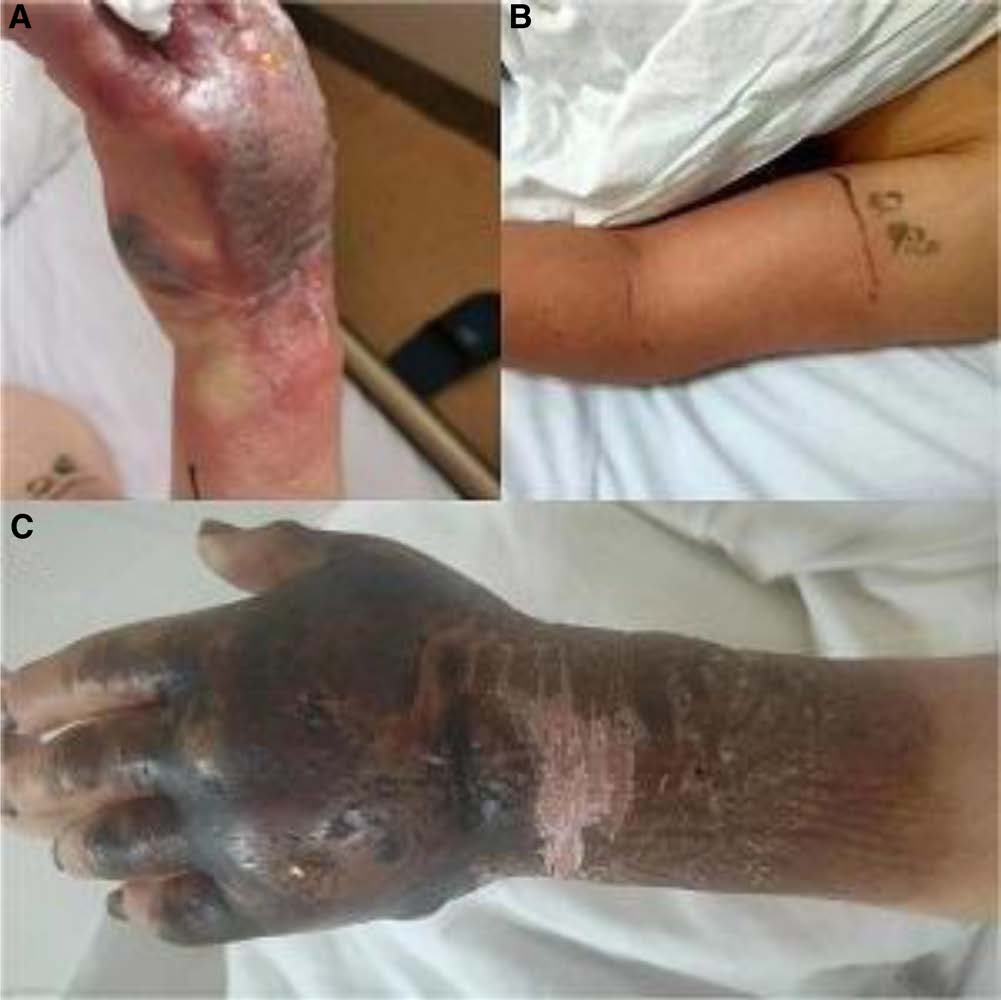
The patient had tension blisters on the back of the left wrist, the back of the hand was slightly dark (A), the left forearm was significantly swollen, and the skin temperature was high (B). The patient’s left hand and the back of his left forearm were black, scabbed, and fell off naturally (C).

The patient was a 63-year-old farmer from Xingtai City, Hebei Province. The main symptoms were local redness and swelling on the back of the left hand for 3 days, with 1 day of fever, prior to being admitted to the Hand Surgery Clinic on May 6, 2022. The patient had a history of contact with an unwell sheep 10 days prior. Following development of the redness and swelling he proceeded to cut it himself. The fever developed 2 days later and the redness and swelling increased, with the left forearm also swelling. On physical examination the patient’s temperature was 36.4°C, pulse 93 beats per minute, obvious swelling of the left hand and forearm, high skin temperature, slightly dark color of the back of the hand, high tension and obvious tenderness, tension blisters on the back of the left wrist, and limited flexion and extension of each finger (Fig. 1A and B). The preliminary diagnosis was cellulitis.

**Figure 2.**
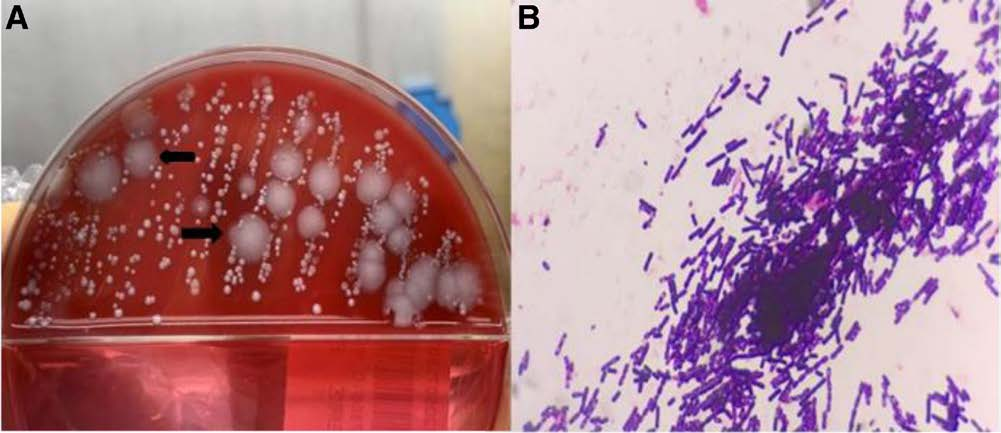
The blister fluid was inoculated onto a blood plate and cultured for 24 hours, white rough colonies appeared, with irregular edges, curly shape, and no hemolysis (arrow in A). Under the microscope, it Gram-stained positive with coarse spore-bearing bacteria (B).

Following admission, blister fluid was extracted for culture. After 24 hours, there were rough colonies with irregular edges, curly shape and no hemolysis, and Gram-stained positive for *Bacillus* under the microscope, suspected of being *B anthracis* (Fig. 2). We conducted mNGS on the extracted vesicular fluid. The DNA nucleic acid was extracted using a QIAamp UCP pathogen minikit (catalog number 50214; Qiagen, Germany). To generate the libraries, a total of 30 μL nucleic acid eluate was used via the Nextera DNA Flex kit (Illumina, San Diego, CA) according to the manufacturer’s instructions, through DNA fragmentation, end repair, adapter ligation and PCR amplification. Then sequenced on an Illumina NextSeq 550 sequencer using a 75-cycle single-end sequencing strategy. For bioinformatics analyses, high-quality sequencing data were generated by removing short (<50 bp), low-quality and human sequence data. The remaining sequencing information was aligned to the most recent databases for bacteria, viruses, fungi, and protozoa (NCBI; ftp:/ftp.ncbi.nlm.nih.gov/genomes). Eighteen sequences of *B anthracis* were detected and the nucleotide homology reached 99.9% with *B anthracis* from the NCBI database (NC_007530.2). We submit the sequences of *B anthracis* to the SRA database in NCBI with the accession number of PRTM1045332 (https://www.ncbi.nlm.nih.gov/sra/PRJNA1045332).

On the second day of admission, the patient was administered 3.2 million units of penicillin every 6 hours, and isolated and closely observed due to the diagnosis of anthrax was established based on mNGS and culturing. On day 3, the patient’s temperature was 38.9 °C, pulse 89 beats per minute, white blood cell count 14.31 × 10^9^/L (normal range 3.5–9.5 × 10^9^/L), neutrophil percentage 83.7% (normal range 40–75%), neutrophil count 11.97 × 10^9^/L (normal range 1.8–6.3 × 10^9^/L), C-reactive protein 170 mg/L (normal range 9–50 mg/L). The heart, lung and abdomen showed no obvious abnormalities, left upper arm redness and swelling had decreased, while other hand symptoms showed no obvious improvement. Treatment was continued. On day 9, the patient had no fever, normal cardiopulmonary and abdominal examinations, slightly swollen left hand and lower forearm, which were dark in color, with a small amount of exudation, and slightly high skin temperature. Alanine aminotransferase was 147 U/L (normal limits 9–50 U/L) and aspartate aminotransferase was 51 U/L (normal limits 15–40 U/L), so compound glycyrrhizin was given to protect the liver, anti-infection treatment continued, potassium permanganate wet applied to the left upper arm, and a brucellosis antibody test completed. On day 18, the patient had no fever, cardiopulmonary and abdominal examination was normal, and the skin on the left hand scabbed and peeled off (Fig. 1C). On day 27, the patient’s condition was stable with slight swelling on the back of his left hand and normal skin temperature.

## 3. Discussion

Anthrax is a zoonotic acute infectious disease, caused by *B anthracis*, prevalent in sheep, cattle, horses, and other herbivores. The main transmission routes are by contact, respiratory tract infection and digestive tract infection.^[8]^ Cutaneous anthrax is the most common form and accounts for >95% of human cases. Humans are infected mainly through contact with infected animals and their products or eating meat from infected animals, which manifests as local skin necrosis and a specific black scab.^[9]^ The patient had a history of contact with an infected sheep 10 days prior to onset of the disease, and the skin lesions of the hand and arm were very similar to the skin lesions of cutaneous anthrax, so anthrax could not be excluded in admission diagnosis.

Cutaneous anthrax is the most common type of anthrax infection; however, clinical cases are still relatively rare, and often have a differential diagnosis with bacterial infectious diseases, such as carbuncle, erysipelas, hemolytic streptococcal gangrene and cellulitis, as well as sheep pox and cowpox. Therefore, definitive identification of the pathogen is required, which in this case was carried out through culture and macro high-throughput genome sequencing. The patient was admitted to the hospital ten days after having had contact with sheep. Three days prior to admission, localized redness and swelling appeared on the back of the left hand, accompanied by 1 day of fever. Upon admission, the patient developed blisters on the dorsal aspect of the left wrist, diagnosed as cellulitis. Fluid from the blisters was sampled for both culture and mNGS. After 24 hours, there were rough colonies with irregular edges, curly shape and no hemolysis, and Gram-stained positive for Bacillus under the microscope. The sequencing results confirmed the presence of 18 sequences of *B anthracis*. Given the inability to rule out anthrax as the diagnosis, the patient was administered 3.2 million units of penicillin every 6 hours. After 1 month of treatment, the patient’s condition stabilized, fever resolved, swelling on the back of the left hand diminished, some skin crusting sloughed off, skin temperature returned to normal, and follow-up indicated a favorable prognosis.

The treatment measures for anthrax are strict isolation, early diagnosis and early treatment. By reading the literatures for the Prevention and Treatment of Anthrax.^[10,11]^ we can know that for nonpregnant adults aged ≥18 years, empiric cutaneous anthrax treatment regimens include choose a single antimicrobial drug or choose a single antitoxin if no antimicrobial drugs are available. The equivalent first-line agents are Doxycycline (100 mg every 12 hours orally), Minocycline (200 mg × 1 dose orally, then 100 mg every 12 hours orally), Ciprofloxacin (500 mg every 12 hours orally), Levofloxacin (750 mg every 24 hours orally), Amoxicillin (1 g every 8 hours orally), and Penicillin VK (500 mg every 6 hours orally). Only choose Amoxicillin and Penicillin VK after the strain has been determined to be penicillin susceptible. Treatment regimen should continue for 7 to 10 days, or until clinical criteria for stability are met. The types, dosage, and duration of medications used by special populations such as pregnant women and children may vary slightly.

Given the long history of successful treatment of localized uncomplicated cutaneous anthrax with penicillin. Therefore, the patient was isolated and treated with penicillin before anthrax was confirmed. The cutaneous anthrax wound should not be squeezed, cut or drained to avoid spread of infection and sepsis. A wet compress with a 1:5000 potassium permanganate solution can be used, or the wound washed with 1:2000 potassium permanganate solution, and antibacterial ointment (such as erythromycin ointment) applied, and then bandaged with sterile gauze.

The limitations in our work lie in the rarity of anthrax in clinical practice. Insufficient awareness or experience among clinicians regarding infections caused by this bacterium can result in misdiagnosis. Distinguishing anthrax from skin infections, cellulitis, and orientia tsutsugamushi is crucial in clinical settings. Fortunately, the application of mNGS technology can serve to compensate for these clinical limitations. It is important to note that Anthrax spores pose a potential bioweapon threat, underscoring the need for stringent biosecurity measures.

With the continuous development of animal husbandry and the increase in cattle and sheep farmers, sporadic cases of cutaneous anthrax have occurred in some parts of China, and the population is generally susceptible. Early symptoms need to be differentiated from other diseases, therefore, clinicians should request detailed medical and contact history, and consider the patient’s place of residence and epidemiological history to avoid misdiagnosis. The mNGS technology can be effectively used to aid early diagnosis. We present the second documented case of cutaneous anthrax successfully diagnosed using mNGS. Traditional bacterial cultures are time-consuming, while mNGS offers the advantage of accurate, quick, high-throughput, unbiased sequencing of all genetic material in a sample.^[12,13]^ This approach, akin to a broad-spectrum net, can detect a wide range of known or unknown pathogens, including *B anthracis*, and boasts a significantly shorter turnaround time. Although the incidence of anthrax has been steadily declining over the past several decades, it still warrants clinical attention.

## Author contributions

**Conceptualization:** Cuiying Zheng, Jiaqing Ye.

**Investigation:** Jiaqing Ye, Minghui Song, Jiahao Hao.

**Methodology:** Cuiying Zheng, Jiaqing Ye.

**Project administration:** Cuiying Zheng, Jiaqing Ye.

**Resources:** Zhongjun Feng, Lijie Zhang.

**Supervision:** Yumei Guo, Weili Gao, Lijie Zhang.

**Validation:** Yumei Guo, Weili Gao, Lijie Zhang.

**Writing – original draft:** Cuiying Zheng, Jiaqing Ye, Minghui Song.

**Writing – review & editing:** Zhongjun Feng, Lijie Zhang.
